# Effect of the Route of Administration of the Vaccinia Virus Strain LIVP to Mice on Its Virulence and Immunogenicity

**DOI:** 10.3390/v12080795

**Published:** 2020-07-24

**Authors:** Sergei N. Shchelkunov, Stanislav N. Yakubitskiy, Alexander A. Sergeev, Alexei S. Kabanov, Tatiana V. Bauer, Leonid E. Bulychev, Stepan A. Pyankov

**Affiliations:** State Research Center of Virology and Biotechnology VECTOR, Rospotrebnadzor, Koltsovo 630559, Novosibirsk Region, Russia; snshchel@vector.nsc.ru (S.N.Y.); sergeev_ala@vector.nsc.ru (A.A.S.); kabanov@vector.nsc.ru (A.S.K.); bauer_tv@vector.nsc.ru (T.V.B.); bulychev@vector.nsc.ru (L.E.B.); pyankov_sa@vector.nsc.ru (S.A.P.)

**Keywords:** smallpox, vaccinia virus, virulence, immunogenicity, vaccination

## Abstract

The mass smallpox vaccination campaign has played a crucial role in smallpox eradication. Various strains of the vaccinia virus (VACV) were used as a live smallpox vaccine in different countries, their origin being unknown in most cases. The VACV strains differ in terms of pathogenicity exhibited upon inoculation of laboratory animals and reactogenicity exhibited upon vaccination of humans. Therefore, each generated strain or clonal variant of VACV needs to be thoroughly studied in in vivo systems. The clonal variant 14 of LIVP strain (LIVP-14) was the study object in this work. A comparative analysis of the virulence and immunogenicity of LIVP-14 inoculated intranasally (i.n.), intradermally (i.d.), or subcutaneously (s.c.) to BALB/c mice at doses of 10^8^, 10^7^, and 10^6^ pfu was carried out. Adult mice exhibited the highest sensitivity to the i.n. administered LIVP-14 strain, although the infection was not lethal. The i.n. inoculated LIVP-14 replicated efficiently in the lungs. Furthermore, this virus was accumulated in the brain at relatively high concentrations. Significantly lower levels of LIVP-14 were detected in the liver, kidneys, and spleen of experimental animals. No clinical manifestations of the disease were observed after i.d. or s.c. injection of LIVP-14 to mice. After s.c. inoculation, the virus was detected only at the injection site, while it could disseminate to the liver and lungs when delivered via i.d. administration. A comparative analysis of the production of virus-specific antibodies by ELISA and PRNT revealed that the highest level of antibodies was induced in i.n. inoculated mice; a lower level of antibodies was observed after i.d. administration of the virus and the lowest level after s.c. injection. Even at the lowest studied dose (10^6^ pfu), i.n. or i.d. administered LIVP-14 completely protected mice against infection with the cowpox virus at the lethal dose. Our findings imply that, according to the ratio between such characteristics as pathogenicity/immunogenicity/protectivity, i.d. injection is the optimal method of inoculation with the VACV LIVP-14 strain to ensure the safe formation of immune defense after vaccination against orthopoxviral infections.

## 1. Introduction

The declaration of global smallpox eradication was solemnly signed at the 33rd World Health Assembly (WHA) on 8 May 1980 [1]. It was the first time in human history that a life-threatening viral disease claiming millions of lives every year was totally eradicated due to the efforts of physicians and scientists from many countries joined under the special World Health Organization (WHO) program [2,3].

A mass vaccination campaign against smallpox has played a crucial role in the eradication of this dangerous disease. Several vaccinia virus (VACV) strains, whose origin was not documented in most cases, were used as a live vaccine in different countries. These VACV strains differed in terms of pathogenicity (when used to infect various species of laboratory animals) and reactogenicity (when used for immunization of humans) [3,4,5,6]. During the mass immunization, all the VACV strains elicited severe adverse reactions in a small percentage of cases, which sometimes resulted in the deaths of people who were being vaccinated. Therefore, a resolution strongly recommending the discontinuation of smallpox vaccination except for investigators at special risk was adopted by the 33rd WHA as early as 14 May 1980 [7].

After the genetic engineering techniques had been developed, VACV started to be used as a molecular vector to design polyvalent vaccines against various infections and as a platform for generating the oncolytic variants of this virus [6]. Genome sequencing of various VACV strains revealed that they are generally similar but have a number of differences (point mutations and small deletions) [3,8,9]. Taking into account that VACV encodes approximately 200 different proteins involved in complex interactions with one another and with proteins of the infected cells, it is not understood yet how the observed differences in DNA sequences of different VACV strains affect their properties. Therefore, each VACV strain and its variants need to be thoroughly studied in different in vivo systems.

The VACV LIVP strain was the study object in the present research. This strain was derived by adapting the Lister strain to calf skin propagation and has been authorized in Russia as the first-generation live smallpox vaccine at the stage of completion of the Global Smallpox Eradication Program. It was shown that LIVP strain was highly immunogenic and, in some cases, more reactogenic for patients than parental VACV Lister [3]. It is known that VACV vaccine strains are genetically heterogenic. Therefore, previously, for genetic engineering reconstruction experiments, we obtained the clonal variant 14 of the LIVP strain (LIVP-14) [10], which was the parental strain in designing the fourth-generation live attenuated smallpox vaccine [11] and the oncolytic VACV variant [12,13,14]. LIVP-14 had the same HindIII endonuclease restriction map for its DNA and growth parameters in cell cultures as parental LIVP strain. The following biological properties of LIVP-14 were studied: neurovirulence after intracerebral injection to neonatal mice, reactogenicity after intradermal (i.d.) injection to rabbits, and immunogenicity after subcutaneous (s.c.) administration to adult mice [10]. 

The pathogenicity and immunogenicity of VACV are known to depend on both the strain being used and the route of its delivery into the animal’s organism [3,4,5]. In this study, we performed a comparative analysis of the virulence and immunogenicity of intranasally (i.n.), i.d., or s.c. administered LIVP-14 to BALB/c mice at different doses, with the purpose of revealing the optimal method of inoculation with the VACV LIVP-14 strain to ensure the safe formation of immune defense after vaccination against orthopoxviral infections.

## 2. Materials and Methods 

### 2.1. Virus

We used clone variant LIVP-14 that was earlier obtained by serial plaque-purifications using agarose overlay [10]. The virus was grown and titrated using the African green monkey kidney cells (CV-1) from the collection of the State Research Center of Virology and Biotechnology (SRC VB) Vector [10]. The monolayer culture of CV-1 cells grown in cell culture flasks using DMEM F-12 medium (Biolot, St. Petersburg, Russia) supplemented with 10% Gibco FBS (Fisher Scientific, Pittsburg, PA, USA) was infected with the virus (multiplicity, 0.1–1.0 pfu/cell) and incubated at 37 °C for 48 h. The infected CV-1 cells were then exposed to two freeze–thaw cycles, and the resulting preparation was subjected to ultrasonic treatment at 22 kHz. The cell debris was flocculated using a J2-HS Beckman Coulter centrifuge (JA-14 rotor, 5000 rpm) at 4 °C for 20 min. The virus was precipitated from the resulting supernatant on a centrifuge at 14,000 rpm at 4 °C for 60 min. The precipitate was resuspended in normal saline (Biolot, St. Petersburg, Russia), placed into 1.5 mL vials, and stored in a deep-freeze refrigerator at −80 °C. Virus titer in the resulting specimens was measured by plaque assay.

### 2.2. Animals

Commonly used for orthopoxviral infections, BALB/c inbred mice (both males and females) were procured from the Laboratory Animals Farm of the SRC VB Vector. The experimental animals were fed a standard diet with a sufficient amount of water, in compliance with the veterinary laws and regulations and the requirements for the humane care and use of laboratory animals. Animal research and manipulations were approved by the Bioethics Committee of SRC VB Vector (permission No 06-09.2019 of 03 Sep. 2019).

Male and female BALB/c mice (age, 3 to 5 weeks; weight, 13–16 g) were used in this study. The mice were i.n. inoculated by the VACV LIVP-14 preparations using the following procedure: a total of 30 µL of virus-containing fluid or normal saline solution (a negative control) was administrated into both nostrils of mice pre-anesthetized with diethyl ether. The mice were infected via the i.d. or s.c. route by a Hamilton syringe injecting the viral preparation or normal saline solution into the tailhead or over the shoulders, respectively, using the same dose and volume of the preparations as for the i.n. inoculation. The infectious doses of 10^8^, 10^7^, and 10^6^ plaque-forming units (pfu) were used. Each group consisted of fifteen experimental animals (six animals for antibody testing and protection experiment, nine animals for virus detection in the internal organs). Six mice of each group were weighed, and external clinical signs of the disease (adynamia, tremor, and ruffled hair coat) were documented daily during 14 days. The experiment was repeated twice. The arithmetic mean mouse body weight in each group at each time point was calculated and expressed as a percentage from the baseline value; the standard deviation of the mean was calculated according to the procedure described in [15].

### 2.3. Virus Detection in the Organs of Infected Mice

The mouse internal organs (lungs, brain, liver, kidneys, and spleen) were collected from mice euthanized by cervical dislocation 3, 7, and 10 days post inoculation (dpi) with viral preparations or normal saline solution. At each time point, organs from three animals were collected and analyzed individually. Organ homogenates (10%) were prepared by mechanical disintegration on a stainless-steel ball homogenizer, with DMEM medium added subsequently. After several freeze–thaw cycles, viral titers in the homogenates were determined on the CV-1 cell culture layer by viral plaque assay.

### 2.4. Sampling Mouse Serum

Twenty-eight days after VACV LIVP-14 preparations or normal saline solution had been administered to mice, blood samples were taken intravitally from the retro-orbital venous sinus using sterile disposable capillaries. Serum was isolated from mouse blood by precipitating blood cells via centrifugation. Individual mouse serum samples were stored at −20 °C.

### 2.5. Enzyme-Linked Immunosorbent Assay of Mouse Serum

Purified VACV LIVP preparation was used as an antigen for ELISA. The antigen (5 × 10^7^ pfu) was adsorbed onto wells of a 96-well ELISA microplate for 18–20 h, and blocking stabilization with 2.5% tryptone enzymatic digest and 5% sucrose was performed for 2 h. The solution had been aspirated from the microplate wells; the microplate was dried and stored in a sealed plastic bag at 4–8 °C. Prior to ELISA, the specimens and reagents under study were kept at room temperature for 30–60 min. A diluting solution (100 µL) consisting of 0.1% casein and 0.1% bovine serum albumin was added to all the microplate wells. Another 100 µL of the diluting solution was added to the top row of the wells. Next, 2 µL of the test specimen was added into each well in the top row; in each microplate, two wells contained two intentionally negative specimens from the control group as a negative control. An intentionally positive sample containing anti-VACV antibodies (SRC VB VECTOR, Koltsovo, Russia) (1:100 dilution) was placed into one microplate well as a positive control.

The test samples and negative control samples (mouse sera with dilutions from 1:100 to 1:12,800) were titrated by pipetting and transferring 100 mL of the solution into the lower rows of the microplate. The wells were sealed with adhesive tape and incubated in a thermoshaker at 37 °C and 700 rpm for 60 min. The excess serum antibodies that had not been bound into immune complexes were removed from the wells by washing five times with 400 µL of washing solution (phosphate buffered saline supplemented with Tween 20 (PBS-T)). A solution of goat anti-mouse IgG (Fc specific)-peroxidase antibody (100 µL) was added to each well; the wells were sealed with adhesive tape and incubated in a thermoshaker at 37 °C and 700 rpm for 30 min. The conjugate that had not been bound to immune complexes was removed from the wells by washing five times with 400 µL of PBS-T. The chromogen 3,3′,5,5′-tetramethylbenzidine in citrate phosphate buffer with hydrogen peroxide (100 µL) was added to each well, and the microplate was incubated at 37 °C for 15 min. The wells in which immune complexes had been formed became blue. The staining reaction was stopped by adding 50 µL of 1 M sulfuric acid (the stop reagent) to each well. The blue color became yellow. The staining intensity was directly proportional to the number of immune complexes formed. The optical density of the solution in the wells was measured on a microplate reader at 450 nm. The anti-VACV IgG titer was calculated by averaging the titers measured for all the replicates for each sample. For each replicate, the titer was quantified if optical density was higher than that of the negative control by at least 10% for the same dilution. The geometric means of log reciprocal titer of VACV-specific IgG were determined for the study groups, and the confidence intervals were calculated for the 95% matching between each sample and the total population.

### 2.6. Determining the Titers of Virus-Neutralizing Antibodies in Serum Samples

The titers of virus-neutralizing antibodies against LIVP VACV strain in mouse serum samples were quantified using the plaque reduction neutralization test (PRNT) according to the decrease in virus plaque count in monolayer cell culture, as described in [16]. Prior to the PRNT, the serum samples were inactivated at 56 °C for 30 min. Fivefold dilution series of serum samples, starting from 1:10 dilution, in the cell maintenance medium were prepared. The dilution where 0.1 mL of the cell culture contained 30–60 pfu was used as the working dilution of VACV. The diluted serum samples and VACV solutions were mixed in equal volume and incubated at 37 °C for 1 h. This mixture (0.2 mL) was placed onto the Vero cell monolayer in 24-well plates; 0.8 mL of the cell maintenance medium was added to each well, and the cells were cultured for 3 days in a CO_2_ incubator. After culturing, the monolayer was stained with a gentian violet solution, and the plaque number in the wells was counted. The geometric means of log titer of VACV-neutralizing antibodies were determined for the study groups, and the confidence intervals were calculated for the 95% matching between each sample and the total population.

### 2.7. Assessment of Protective Immune Response in Mice Infected with LIVP-14

On 29 dpi, a preparation of the cowpox virus strain GRI-90 (CPXV-GRI) [17] at a dose of 32 LD_50_ (30 µL) was i.n. administered to mice that had previously been infected with LIVP-14 or had received saline solution. The mice were followed up for 14 days, and deaths were documented.

### 2.8. Statistics

Statistical treatment of results was carried out with standard methods using the software package Statistica 6.0 (StatSoft, Tulsa, OK, USA), with assessment of significant differences (*p* < 0.05) for a 95% confidence level [18].

## 3. Results

### 3.1. The Pathogenicity of VACV LIVP-14 Depends on the Route of Its Administration to Mice

In order to perform a comparative study to evaluate the effect of the route of infection and dose of the administered viral preparation on the pathogenicity of the VACV LIVP-14 strain, mice were infected via the three most popular routes: i.n., i.d., or s.c. The infective doses were 10^6^, 10^7^, or 10^8^ pfu/animal. Since inoculation with most VACV strains usually does not cause death in adult mice, the pathogenicity of variants of this virus is studied according to changes in animals’ body weight after the infection and clinical manifestations of the disease (ruffled hair coat, adynamia, and tremor) [15,19].

**Figure 1:**
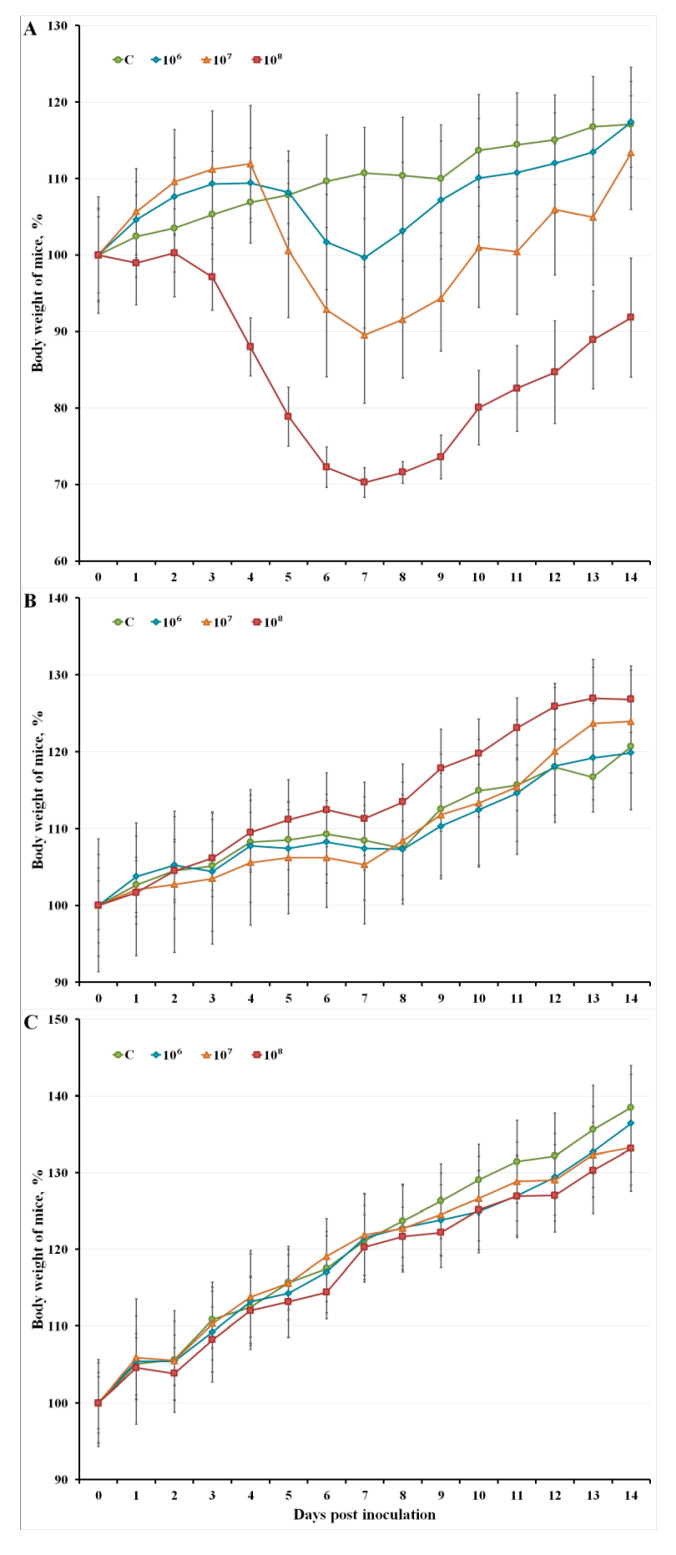
Changes in mouse body weight after intranasal (**A**), intradermal (**B**), and subcutaneous (**C**) inoculation with VACV LIVP-14 (doses of 10^6^, 10^7^, and 10^8^ pfu) or saline solution (C, control). The error bars indicate the standard deviation of the mean of mouse body weight in each group consisting of six animals.

Pronounced clinical manifestations of infection were observed after i.n. administration of LIVP-14 to mice starting on day 3. The increasing virus dose led to more obvious clinical signs of the infection and more significant body weight loss in mice (Figure 1A). The peak of the disease occurred on 6–8 dpi.

No clinical manifestations of the disease were observed in i.d. and s.c. infected mice with LIVP-14; no statistically significant changes in body weight compared to the control group animals that received saline solution were observed (Figure 1B,C).

### 3.2. Dissemination of VACV-14 to Mouse Organs

The organs of i.n., i.d., or s.c. infected animals with LIVP-14 at doses of 10^6^, 10^7^, or 10^8^ pfu/animal and removed on 3, 7, and 10 dpi were used to prepare 10% homogenates. Viral titers were determined using the plaque assay on the CV-1 cell culture. Lung, brain, liver, spleen, and kidney samples were analyzed.

**Figure 2:**
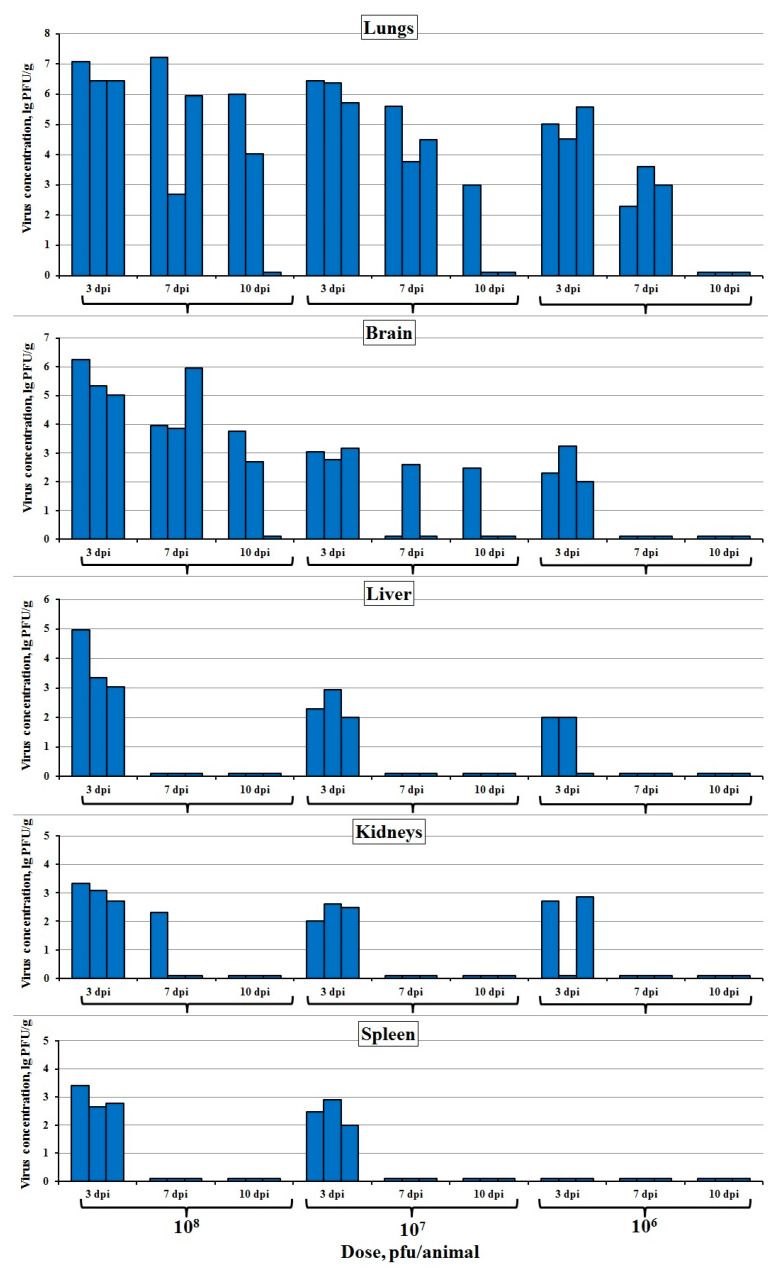
VACV concentration in the internal organs of mice intranasally infected with the LIVP-14 strain at doses of 10^6^, 10^7^, and 10^8^ pfu. The data for individual animals on days 3, 7, and 10 post-inoculation (dpi) are presented. Organs of three mice were analyzed at each time point.

The i.n. administered virus LIVP-14 replicated efficiently in the lungs (Figure 2). The lung titer of LIVP-14 gradually decreased from 3 dpi to 10 dpi. On day 10, it was detected in two of three animals for the infective dose of 10^8^ pfu, in one of three animals for the infective dose of 10^7^ pfu, and in none of the three studied animals for the infective dose of 10^6^ pfu. It should be mentioned that the lower limit of detection under our experimental conditions was 10^2^ pfu/g.

When the virus was i.n. inoculated at a high infective dose, it was efficiently accumulated in the brain and was detected in it on 3, 7, and 10 dpi, while being observed in the liver, kidneys, and spleen only on 3 dpi (Figure 2).

After s.c. administration, LIVP-14 was revealed in none of the samples of the internal organs under study. The virus was detected only in the skin flap at the inoculation site. 

**Figure 3:**
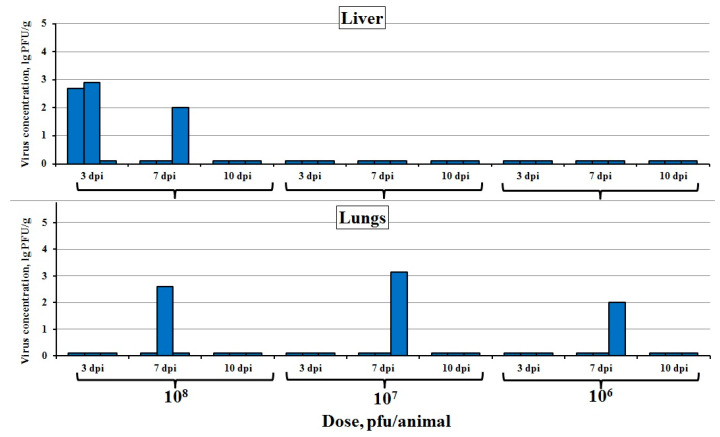
VACV concentration in the internal organs of mice intradermally infected with the LIVP-14 strain at doses of 10^6^, 10^7^, and 10^8^ pfu. The data for individual animals on days 3, 7, and 10 post-inoculation (dpi) are presented. Organs of three mice were analyzed at each time point.

The i.d. injected virus at a dose of 10^8^ pfu was detected in the liver in some animals on 3 and 7 dpi. In one-third of the animals, LIVP-14 was revealed in the lungs on 7 dpi for all three infective doses (Figure 3). After i.d. administration, LIVP-14 was not revealed in the samples of the other internal organs under study.

### 3.3. The Immunogenicity of VACV Depends on the Route Used for Its Inoculation to Mice

The immunogenicity of VACV LIVP-14 was assessed according to the level of virus-specific (ELISA) and virus-neutralizing (PRNT) antibodies in mouse serum samples obtained on 28 dpi, using three different methods of inoculation with several doses of the virus (10^6^, 10^7^, or 10^8^ pfu/animal).

**Figure 4:**
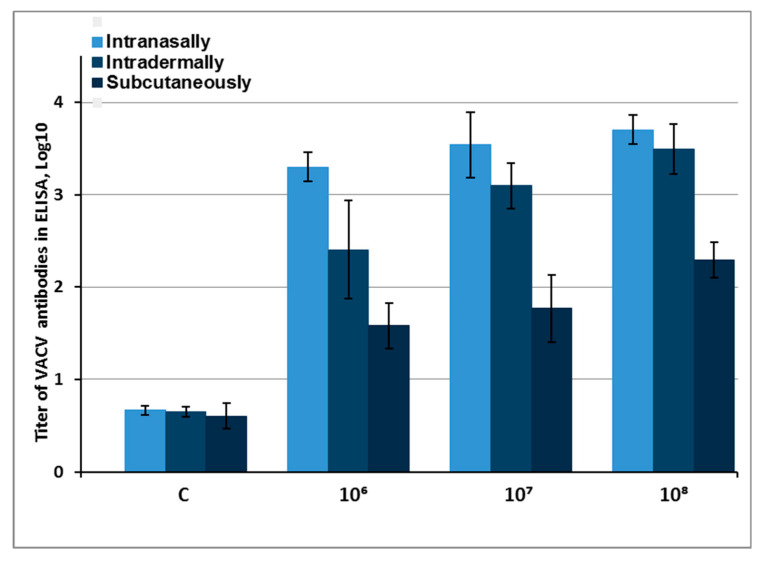
Concentrations of VACV-specific IgG antibodies determined by ELISA in serum samples of mice infected with different doses (10^6^, 10^7^, and 10^8^ pfu) of the LIVP-14 strain via different routes. C (control)—serum samples from mice that received saline solution. The error bars indicate 95% lower and upper limits of the estimated titer. Each group consisted of six animals.

The results of ELISA quantifying the titers of IgG antibodies against antigens of purified VACV LIVP virions in these serum samples show that the highest level of production of virus-specific antibodies was observed for i.n. infected mice (Figure 4). The lowest level of biosynthesis of antibodies against VACV virion antigens was observed after s.c. inoculation with the virus. After i.d. injection with LIVP-14, the level of virus-specific antibodies was appreciably high; at infective doses of 10^8^ and 10^7^ pfu, it did not differ significantly from the respective antibody levels in i.n. infected mice.

**Figure 5:**
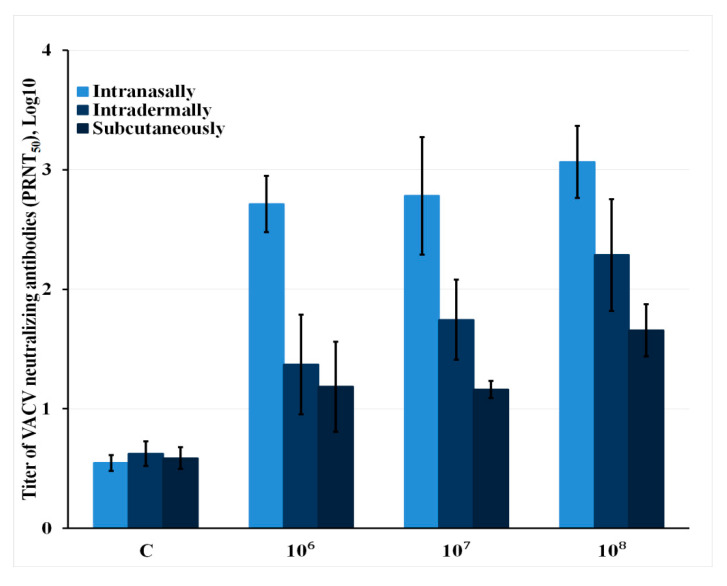
The data on VACV-neutralizing activity of serum samples collected on day 28 after the mice had been infected with different doses (10^6^, 10^7^, and 10^8^ pfu) of the LIVP-14 strain via different routes. C (control)—serum samples from mice that received saline solution. The error bars indicate 95% lower and upper limits of the estimated titer. Each group consisted of six animals.

An analysis of the level of virus-neutralizing antibodies showed a picture that was generally similar to the ELISA data but also had some differences (Figure 5). The highest production of virus-neutralizing antibodies was revealed for i.n. administered VACV LIVP-14. The weakest immune response was observed for s.c. injection of the virus. When LIVP-14 was i.d. inoculated at infective doses of 10^8^ and 10^7^ pfu/animal, the synthesis level of neutralizing antibodies was relatively high but statistically lower than that of i.n. infected mice.

### 3.4. Protectivity of the Immune Response in Mice Infected with LIVP-14

On 29 dpi after the i.n. or i.d. infection with the LIVP-14 at a dose of 10^6^ or 10^7^ pfu, the protectivity of the immune response to the orthopoxviral infection in mice was analyzed. The animals were i.n. inoculated with CPXV-GRI at a dose of 32 LD_50_ and were followed up for 14 days. Results showed that, regardless of the dose of the LIVP-14 virus and the two aforementioned routes used for its inoculation, mice developed an immune response ensuring 100% defense against CPXV infection. In the control group, all animals died by 8 dpi with CPXV. Due to the low antibody response, mice after s.c. infection were not analyzed in the protectivity test.

## 4. Discussion

The method proposed by E. Jenner for immunoprevention of smallpox in the late 18th century consisted in inoculation of humans with infectious material taken from skin lesions formed when animals or humans were infected with cowpox or horsepox [20]. Back then, the nature of infectious agents of cowpox, horsepox, and smallpox was unknown, since it was not until one century later that the kingdom of viruses was discovered. As a result, the vaccinating agents against smallpox widely used across different world regions for many years were not clearly characterized. Meanwhile, they had undergone multiple replication cycles/passages in the body (typically skin) of various animals such as calves, rabbits, or sheep, so the variants exhibiting lower reactogenicity were selected for the vaccination of humans. Therefore, different strains of vaccine viruses were historically used in different geographic regions. Later on (in the 20th century), these viruses were classified as belonging to the species *Vaccinia virus* (VACV), which differs from the causative agent of cowpox, the *Cowpox virus* (CPXV) [6,20]. Both these species are closely related to the smallpox virus, whose strains form the species *Variola virus* (VARV) and are members of the genus *Orthopoxvirus* in the family Poxviridae [3]. Orthopoxviruses are antigenically and immunologically related, they elicit serological cross-reactivity, and they provide an immune defense in mammals infected with a related virus species. For this very reason, the sporadic infection of humans with the CPXV or vaccination with VACV protected them against smallpox [2,3].

During the international program of global smallpox eradication, which was started under the auspices of the WHO in 1958 and was completed in 1980, a number of VACV strains most widely used for vaccination were compared in terms of such parameters as reactogenicity, postvaccinal complications after immunization of humans, and pathogenicity for laboratory animals (mice and rabbits). A correlation was revealed for these properties between the clinical data and results of animal studies [3]. It was found that the VACV strains can differ significantly in terms of their pathogenicity and reactogenicity. Furthermore, the route of inoculation with the virus can also significantly affect its pathogenic and immunogenic properties [21,22,23,24,25,26].

The study object in the present work was the LIVP strain generated at the Institute of Viral Preparations (Moscow, Russia) by adapting the Lister VACV strain to calf skin propagation and authorized for producing the first-generation smallpox vaccine in Russia [3]. Before genetic engineering experiments, we have obtained and partially characterized the clonal variant LIVP-14 of this VACV strain [10]. In this study, we evaluated the effect of the dose and route of administration of this virus on its pathogenicity and immunogenicity in the mouse model of the infection caused by it.

Several methods of smallpox vaccination were used, all involving the introduction of VACV into Malpighian layer of the epidermis, with a variety of instruments. In most countries, either the skin was scarified by a single linear incision or scratch, or the vaccine was introduced into the epidermis by the multiple pressure method [2]. In our study, we used i.d. VACV administration as an analogous laboratory technique for vaccination in a mice model.

Adult mice exhibited the highest sensitivity to the i.n. inoculated LIVP-14 strain, although the infection did not lead to animal death. For this route of inoculation, pronounced clinical manifestations of the infection (adynamia, tremor, and ruffled hair coat) were observed, starting on 3 dpi. More obvious clinical signs of infection and greater body weight loss were observed with increasing virus dose (Figure 1A). Additionally, i.d. or s.c. injection with the same infective doses of LIVP-14 did not result in clinical signs of the disease, and the changes in animals’ body weight were similar to those in control animals (Figure 1B,C).

The i.n. inoculated LIVP-14 replicated efficiently in the lungs (Figure 2). Moreover, this virus was accumulated in the brain at relatively high concentrations. Lower levels of LIVP-14 were detected in the liver, kidneys, and spleen of the experimental animals (mainly on 3 dpi). Accumulation of the virus in the internal organs indicates that, after i.n. administration, it is efficiently disseminated in the mouse body. The differences in VACV concentration in various organs correlate with the variations in infective virus doses.

When LIVP-14 was s.c. inoculated, it was detected in none of the samples of the internal organs under study. The virus was revealed only in the skin flap at the inoculation site. This fact demonstrates that this VACV strain replicated locally at the inoculation site even at high infective doses.

After an i.d. injection at a dose of 10^8^ pfu, the virus was detected in the liver in some animals on 3 and 7 dpi. In one-third of the animals, LIVP-14 was detected in the lungs on 7 dpi for all the infective doses (Figure 3). There were no detectable virus titers in the brain, spleen, and kidneys. Hence, unlike the s.c. route, the i.d. injection of VACV LIVP-14 leads to the limited distribution of this virus within the mouse body and does not cause pronounced clinical manifestations.

It is known that the level of antibody-mediated immune response to vaccination against orthopoxviral infections plays a decisive role in protection against the subsequent viral infection [20,27,28,29]. Therefore, in this study we have limited ourselves to investigating the induction of biosynthesis of antiviral antibodies depending on the route and dose of inoculation of LIVP-14 in laboratory mice. An analysis of the level of antiviral antibodies in serum samples from mice infected with the VACV LIVP-14 strain under study was performed using two methods. ELISA was used to test the antibodies specifically interacting with VACV virion proteins. PRNT was used to quantify the concentrations of antibodies in the same serum samples, which were in vitro binding to the virions and inhibited their infectivity (plaque formation) in the cell culture.

The results of ELISA for serum samples from the infected mice demonstrated that the highest level of production of VACV-specific antibodies was observed in i.n. infected mice (Figure 4). Moreover, s.c. inoculation of the virus led to the statistically significantly lowest biosynthesis of antibodies against VACV virion antigens. Upon i.d. inoculation of LIVP-14, the level of specific antibodies was high and comparable to the concentration of VACV-specific IgG in the serum from i.n. infected mice with infective doses of 10^7^ and 10^8^ pfu.

When the level of virus-neutralizing antibodies was studied using the PRNT, a regularity similar to that of the ELISA data but with certain differences was obtained (Figure 4 and Figure 5). The highest production of virus-neutralizing antibodies was observed for i.n. inoculation with VACV. The weakest immune response was detected after s.c. injection of the virus. After i.d. administration of LIVP-14, the synthesis level of neutralizing antibodies was relatively high, while being statistically significantly lower compared to concentration of VACV-neutralizing antibodies in i.n. infected mice. These differences in the ratio between the levels of antiviral antibodies in i.n. and i.d. inoculated mice with VACV LIVP-14 revealed by ELISA and PRNT seem to demonstrate that a significant portion of virion-specific IgG antibodies synthesized in response to LIVP infection are not virus-neutralizing in the test used in this study.

One day after blood samples had been taken to analyze the level of VACV-specific antibodies (29 dpi), mice that were i.n. or i.d. inoculated with LIVP-14 at doses of 10^6^ or 10^7^ pfu were tested to assess the protectivity of the elicited immune response to the lethal orthopoxviral infection. Results showed that, regardless of the doses of the LIVP-14 virus used in this study and the two routes of its inoculation mentioned above, mice developed an immune response yielding 100% protection against infection with the CPXV-GRI virus at a dose of 32 LD_50_. In the control groups, all animals died by 8 dpi with CPXV. Due to low antibody response, mice after s.c. infection were not analyzed in the protectivity test.

Our findings imply that, according to the ratio between such characteristics as pathogenicity/immunogenicity/protectivity, i.d. injection is the optimal method of inoculation with the VACV LIVP-14 strain to ensure the safe formation of immune defense after vaccination against the orthopoxviral infections.
